# Evaluation of effectiveness and safety of the multizone NeVa^TM^ stent retriever for mechanical thrombectomy in ischemic stroke

**DOI:** 10.1007/s00234-023-03236-4

**Published:** 2023-10-25

**Authors:** Max Masthoff, Hermann Krähling, Burak Han Akkurt, Mohamed Elsharkawy, Michael Köhler, Mostafa Ergawy, Christian Thomas, Wolfram Schwindt, Jens Minnerup, Paul Stracke

**Affiliations:** 1 Clinic for Radiology, University of Muenster and University Hospital Muenster, Albert-Schweitzer-Campus 1, 48149 Muenster, Germany; 2 Clinic for Radiology and Neuroradiology, Alfried Krupp Hospital, Essen, Germany; 3 Institute of Neuropathology, University of Muenster and University Hospital Muenster, Muenster, Germany; 4 Department of Neurology with Institute of Translational Neurology, University of Muenster and University Hospital Muenster, Albert-Schweitzer-Campus 1, 48149 Muenster, Germany; 5 Clinic and Policlinic for Diagnostic and Interventional Neuroradiology, University Hospital Hamburg-Eppendorf, Hamburg, Germany

**Keywords:** Acute ischemic stroke, Mechanical thrombectomy, Multizone stent retriever

## Abstract

**Purpose:**

This study aimed to evaluate the effectiveness and safety of the NeVa^TM^ stent retriever as first- and second-line device for mechanical thrombectomy in acute ischemic stroke.

**Methods:**

In this retrospective single-center study, all consecutive patients that underwent mechanical thrombectomy with NeVa^TM^ stent retriever as first- or second-line device due to intracranial vessel occlusion with acute ischemic stroke between March and November 2022 were included.

**Results:**

Thirty-nine patients (m=18, f=21) with a mean age of 69.9 ± 13.3 years were treated with the NeVa^TM^ stent retriever. NeVa^TM^ stent retriever was used as first-line device in 24 (61.5%) of patients and in 15 (38.5%) as second-line device. First-pass rate (≥mTICI 2c) of NeVa^TM^ stent retriever was both 66.7% when used as first- or second-line device. Final recanalization rate including rescue strategies was 92.3% for ≥mTICI2c and 94.9% for ≥mTICI2b. No device-related minor or major adverse events were observed. A hemorrhage was detected in 33.3% of patients at 24h post-thrombectomy dual-energy CT, of which none was classified as symptomatic intracerebral hemorrhage. NIHSS and mRS improved significantly at discharge compared to admission (*p*<0.05).

**Conclusion:**

The NeVa^TM^ stent retriever has a high effectivity and good safety profile as first- and second-line device for mechanical thrombectomy in acute ischemic stroke.

## Introduction

Mechanical thrombectomy (MT) based on clot-retrieval by stent retrievers, perceived as the most effective devices for fast and safe recanalization superior to intravenous thrombolysis alone, has emerged as standard care for patients with acute ischemic stroke (AIS) [1–5]. MT aims to accomplish complete reperfusion of occluded vasculature; herein, the achieved grade of recanalization is measured by the modified Thrombolysis in Cerebral Infarction (mTICI) Score. Successful recanalization defined as mTICI≥2b as a measure for technical success was used in the majority of studies [6]. Meanwhile, it has been shown that higher reperfusion rates and especially complete recanalization (mTICI 3) lead to improved clinical outcome [7]. Fast recanalization as measured by the first-pass rate (complete reperfusion ≥mTICI 2c with a single pass of a stent retriever) has also been shown to be associated with significantly higher rates of good clinical outcome and is therefore used to evaluate the performance of new devices [8–10]. In recent years, various new stent retrievers with different size, shapes, and materials have been introduced. While the choice of material is currently up to the neurointerventionalists’ discretion depending on the individual case, evidence-based decision-making should be fostered by future research. In this context, it has been shown for some devices that a larger diameter or longer size of the stent retriever may improve successful first-pass rate and final mTICI score in large vessel occlusion [11, 12]. However, MT may still remain challenging especially with organized or hard, fibrin-rich, and sticky clots. The NeVa^TM^ stent retriever device has recently been designed with multifunctional drop zones and high radial force to improve first-pass rate for all clot types. The NeVa^TM^ stent retriever device showed promising results in first preclinical [13, 14] and clinical studies [15–19], but evidence for first- and especially second-line use of this device is still limited.

The aim of this study was therefore to evaluate the effectiveness and safety of the NeVa^TM^ stent retriever as first- and second-line device for mechanical thrombectomy in acute ischemic stroke.

## Methods and material

### Study design

This retrospective study included all consecutive patients with acute ischemic stroke (AIS) who received mechanical thrombectomy (MT) with the use of the NeVa^TM^ 4 × 30-mm stent retriever (Vesalio LLC, Nashville, USA) in a tertiary stroke center between March and November 2022. The study was approved by the local ethics committee. Informed patient consent was waived due to the retrospective character of this study.

Diagnosis of AIS was made by computed tomography (CT), CT angiography (CTA), and CT perfusion (CTP), if patient presented in-house. Patients transferred from external hospitals were examined there by CT or magnetic resonance imaging (MRI) and referred to the study center depending on imaging results and supply capacity. Additional intravenous lysis therapy was performed immediately after diagnosis in case of no contraindications. Neurological assessments were performed by trained neurologists in a tertiary stroke center. Decision for mechanical thrombectomy was made according to national guidelines [20] and in consensus by interventional neuroradiologist and neurologist on service, having at least 5 years of professional experience in stroke care.

Baseline data of the study cohort were retrieved from the Clinical Information System (CIS) and the radiology information system (RIS) as well as the Picture Archiving and Communication System (PACS) regarding:

**•** Patient characteristics (sex; age)

**•** Baseline clinical parameters (modified Rankin scale (mRS); National Institutes of Health Stroke Scale (NIHSS); etiology of stroke; preprocedural intravenous lysis therapy; time from symptom onset to femoral puncture)

**•** Baseline imaging characteristics (location and side of vessel occlusion)

*Mechanical thrombectomy: technique and technical outcome evaluation*

**Figure 1:**
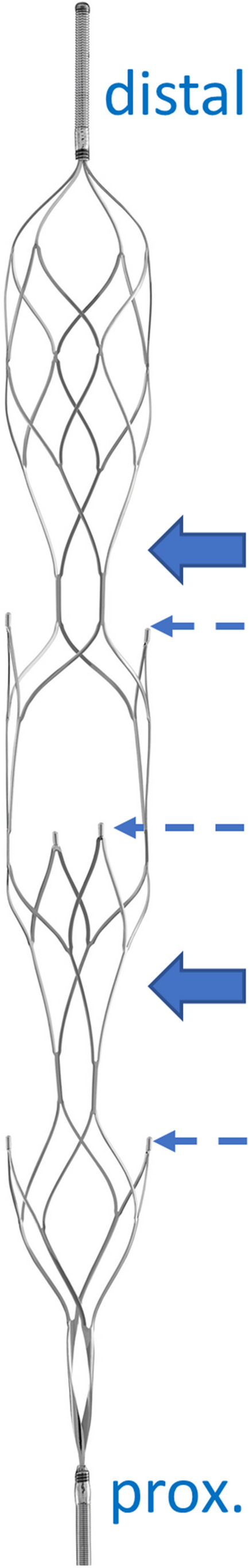
Design of NeVa^TM^ stent retriever. Illustration of the NeVa^TM^ stent retriever (4 × 30 mm) consisting of two drop zones (blue bold arrows) labeled by radiopaque markers (blue dotted arrows). The drop zones are designed as entry points for clots. The closed-ended basket-shape zone at the distal end is supposed to retain entrapped thrombus (photograph provided by Vesalio LLC, Nashville, USA)

**Figure 2:**
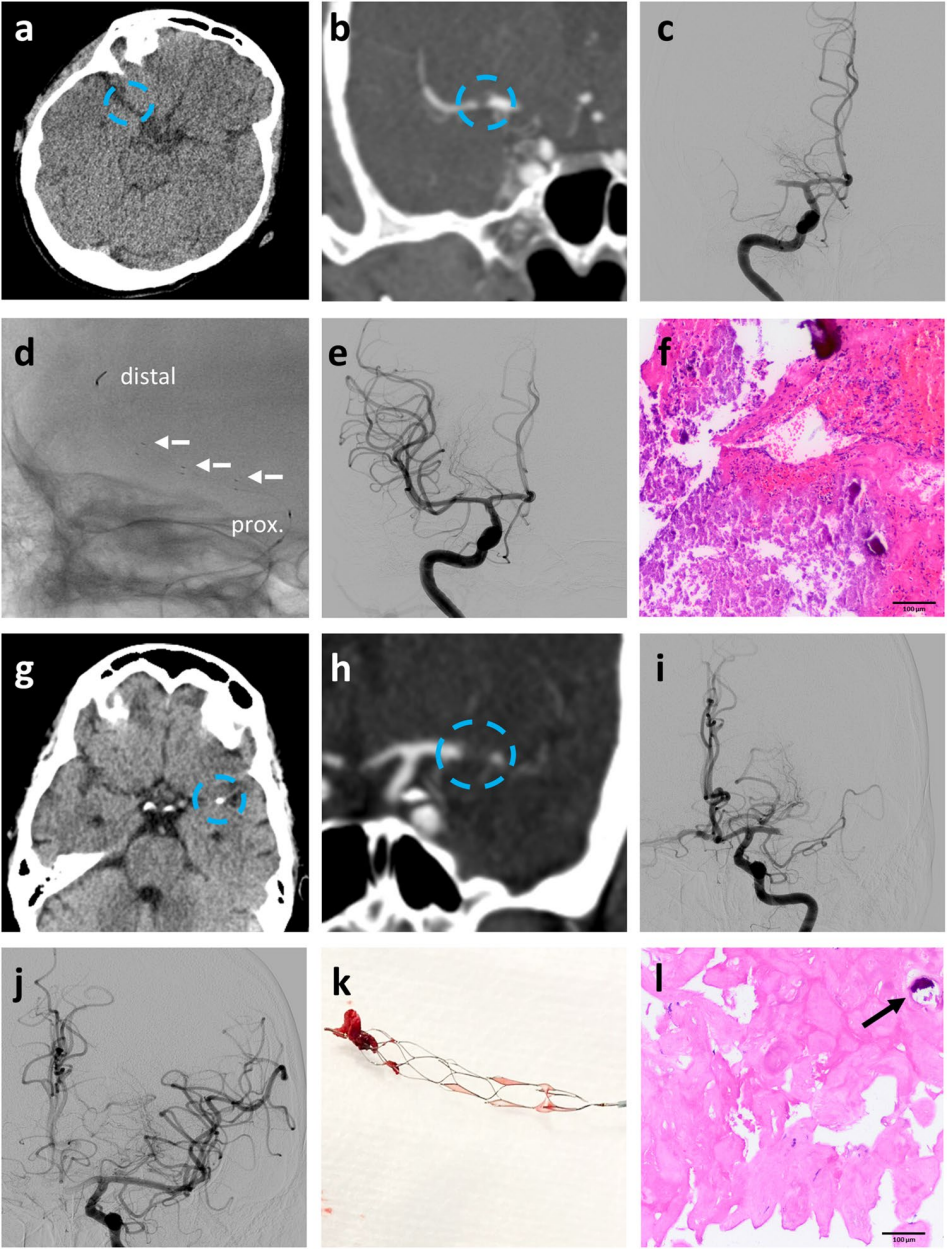
Exemplary case examples of mechanical thrombectomy with NeVa^TM^ stent retriever in mechanical thrombectomy with different thrombus morphology. **a** Axial CT and **b** coronal CT angiography of a patient with acute ischemic stroke due to *hypodense* thrombus (blue circle) in M1 segment of the right middle cerebral artery. **c** Digital substraction angiography (DSA) showed right M1 occlusion. **d** Placement of NeVa^TM^ stent retriever at the site of M1 occlusion, radiopaque markers (proximal, distal, and drop zones (white arrows)) are clearly depicted. **e** DSA after one pass with NeVa^TM^ stent retriever showed complete recanalization of the previously occluded segment. **f** Histology of retrieved thrombus showed mainly erythrocyte-rich areas with only some fibrin and calcifications, correlating with CT findings. **g** Axial CT and **h** coronal CT angiography of a patient with acute ischemic stroke due to *hyperdense*, calcified thrombus (blue circle) in M1 segment of the left middle cerebral artery. **i** Digital substraction angiography (DSA) showed left M1 occlusion. **j** DSA after one pass with NeVa^TM^ stent retriever showed complete recanalization of the previously occluded segment. **k** Photograph of a thrombus of a different patient retrieved by NeVa^TM^ stent retriever showing entrapped thrombus at distal end of the device. **l** In contrast to patient **a**–**f** histology of the thrombus retrieved from the patient **g**–**j** showed a heavily fibrin-rich, low in erythrocytes thrombus with some calcifications (black arrow) correlating with CT data. As shown by patients **a**–**f** and **g**–**l**, NeVa^TM^ showed successful first-pass recanalization in both types of thrombus causing acute cerebral ischemia

MT was performed by trained and certified interventional neuroradiologists with at least 5 years of professional experience. In all cases, MT was performed via an arterial transfemoral approach in general anesthesia. This retrospective study included patients with use of NeVa^TM^ stent retriever as first- and second-line device for MT. Thus, before- or afterhand use of other stent retriever devices was not an exclusion criterion. The choice to use NeVa^TM^ stent retriever as first- or second-line device was left to the discretion of the neurointerventionalist performing the procedure and depended on the location of the thrombus as well as underlying vessel size and configuration. The NeVa^TM^ stent retriever has been described in detail elsewhere [13, 16]. Briefly, the device is a novel hybrid-cell, multizone stent retriever consisting of two large open areas ensuring entry points for clots as well as a closed-ended basket-shape zone at the distal end retaining entrapped thrombus (Fig. 1). The design is supposed to retrieve both CT hypodense (Fig. 2a–f) and CT hyperdense, fibrin-rich/calcified thrombus (Fig. 2g–k). In all cases, either an 8F balloon-guided catheter (FlowGate, Stryker, Kalamazoo, USA) or 6F long sheath (NeuronMax, Penumbra, Alameda, USA) was placed in the internal carotid artery, connected with a continuous flush line charged with nimodipine (2 mg/l in saline). Intermediate catheters, such as ACE68, JET7, 5MAX (Penumbra, Alameda, USA) or 5F Sofia, and REACT68 (Medtronic, Irvine, USA), were also used at the discretion of the performing neurointerventionalist. A microcatheter (Rebar18, Medtronic, Irvine, USA) was placed distal to the occluding thrombus and the stent retriever deployed. Withdrawal of the stent retriever was performed under suction by a 50cc syringe or electric aspiration pump at the guiding catheter and, if used, the intermediate catheter.

Procedural data analysis included if the procedure was performed with or without balloon-guided catheter as well as with or without intermediate catheter, usage of the NeVa^TM^ stent retriever as first- or non-first-line device, rate of stent retriever changes from NeVa^TM^ to other stent retrievers and vice versa, and number of passes with NeVa^TM^ stent retriever.

Technical outcome evaluation was based on the Modified Thrombolysis in Cerebral Infarction (mTICI) [21] score with NeVa^TM^ first-pass ≥mTICI 2c and ≥mTICI 2b recanalization rate and final mTICI score. Technical outcome evaluation was performed by board certified radiologists with at least 5 years of professional experience in stroke care.

### Mechanical thrombectomy: safety evaluation

All procedural data and patient recordings were analyzed with regard to any device-related minor or major adverse events. Further, postinterventional dual-energy CT data performed 24 h after MT were reviewed for any intracranial hemorrhage. Symptomatic intracerebral hemorrhage (SICH) was defined based on ECASS-III (European Cooperative Acute Stroke Study) criteria as any intracranial hemorrhage associated with neurological deterioration of ≥4 points on the NIHSS score at 24 h [22]. Safety outcome evaluation was performed by board certified radiologists (CT) and stroke neurologists (SICH) with at least 5 years of professional experience in stroke care.

### Clinical outcome evaluation

Clinical outcome after MT was based on the extent of neurological impairment as assessed by NIHSS at 24h after MT as well as NIHSS and mRS at the time of discharge. Neurological assessments for clinical outcome evaluation were performed by trained stroke neurologists of a tertiary care stroke center.

### Statistical analysis

Statistical analyses were performed using SPSS version 28.0.1 (SPSS Inc., Chicago, IL, USA). All data are presented as the mean (±SD), median (range), absolute, or percentage depending on nature of variables and distribution. Chi-square test was used for contingency tables. For analysis of any parameters associated with a successful first-pass recanalization of the NeVa^TM^ stent retriever (≥mTICI 2c), a binary logistic regression was used. For comparison of NIHSS at admission, 24h after MT and at discharge a Friedman test for non-parametric paired data with respective post hoc test was used. For comparison of mRS score at admission and discharge, a paired *t*-test was used. Two-sided *p*-values < 0.05 were defined as statistically significant.

## Results

### Study cohort

This study included 39 patients (18 male, 21 female) with a mean age of 69.9 (± 13.3, range 26–88) years who received a mechanical thrombectomy with NeVa^TM^ stent retriever, selected from a total cohort of 212 patients receiving mechanical thrombectomy within the study period. A total of 25 (64.1%) patients presented with vascular occlusion of the M1 segment of the middle cerebral artery (MCA-M1), 7 (17.9%) patients with occlusion of the M2 segment of the middle cerebral artery (MCA-M2), 3 (7.7%) patients presented with a combined occlusion of the internal carotid artery (ICA) and an intracranial vessel (2× M1, 1× M2), and 4 (10.3%) patients presented with occlusion of the basilar artery (BA). Vessel occlusions of anterior circulation were located on the right side in 22 (56.4%) and on the left side in 13 (33.3%) of patients. Systemic lysis therapy was performed in 15 (38.5%) patients. The mean time from onset of symptoms to femoral puncture was 297.8 ± 203.6 min. Etiology of stroke was cardioembolic in 12 (30.8%) patients, thrombotic due to local stenosis in 2 (5.1%) patients, due to aortic valve endocarditis in 1 (2.6%) patient, embolic after thoracic surgery in 1 (2.6%) patient, due to drug abuse in 1 (2.6%) patient, and unknown in 22 (56.4%) patients.

Detailed patient characteristics are shown in Table 1.

**Table 1:** Patient characteristics

Parameter	*n* (%)	p-value*
All patients	39 (100.0)	
Age (mean ± SD)	69.9 ± 13.3	0.114
Sex		0.350
Male	18 (46.2)	
Female	21 (53.8)	
Location of vessel occlusion		0.051
M1	25 (64.1)	
M2	7 (17.9)	
ICA + intracranial vessel	3 (7.7)	
BA	4 (10.3)	
Right-sided	22 (56.4)	
Left-sided	13 33.3)	
Etiology of stroke		0.632
Cardioembolic	12 (30.8)	
Thrombotic	2 (5.1)	
Endocarditis	1 (2.6)	
Thoracic surgery	1 (2.6)	
Drug abuse	1 (2.6)	
Unknown	22 (56.4)	
Systemic i.v. lysis therapy		0.150
Yes	15 (38.5)	
No	24 (61.5)	
Time from symptom onset to femoral puncture (mean ± SD in min)	297.8 ± 203.6	0.433

Abbreviations: *BA* basilar artery; *i.v.* intravenous; *M1* M1-segment of middle cerebral artery; *M2* M2-segment of middle cerebral artery; *min* minutes; *SD* standard deviation; *p-values are given for binary logistic regression for respective baseline parameters regarding first-pass recanalization mTICI≥2c with NeVa^TM^ stent retriever

### Mechanical thrombectomy: procedural characteristics and technical outcome evaluation

**Figure 3:**
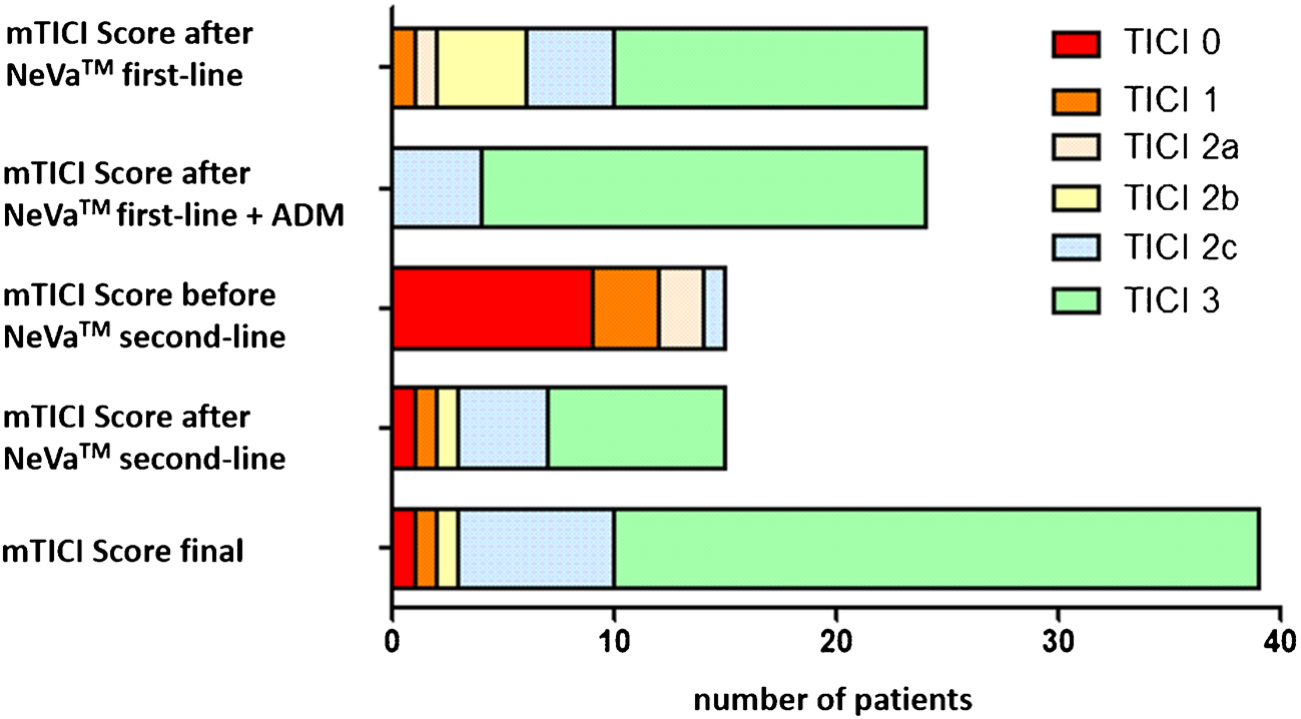
mTICI scores of first- or second-line NeVa^TM^ stent retriever use. mTICI scores 1) after NeVa^TM^ stent retriever as first-line device 2), after NeVa^TM^ stent retriever as first-line device + additional maneuvers (ADM; other devices, rescue etc.) 3), before NeVa^TM^ stent retriever as second-line device 4), after NeVa^TM^ stent retriever as second-line device, and 5) final mTICI score of the entire cohort

Detailed information on procedural characteristics and technical outcome are shown in Table 2 and recanalization rates are also illustrated in Fig. 3. The NeVa^TM^ stent retriever was used as first-line thrombectomy device in 24 (61.5%) patients and in 15 (38.5%) patients as second-line device. A balloon-guided catheter was used in 30 (76.9%) patients; an intermediate catheter was used in 13 (33.3%) patients. Additional stenting was performed in 4 (10.3%) patients due to underlying stenosis (75%) or dissection (25%, not NeVa^TM^ stent retriever related).

**Table 2:** Procedural characteristics and outcome

Parameter	*n* (%)	p-value*			
NeVa^TM^ stent retriever as					
First-line device	24/39 (61.5)	0.350*			
Second-line device	15/39 (38.5)				
No of passes	NeVa^TM^ first-line	Before NeVa^TM^ second-line	NeVa^TM^ second-line	Total (NeVa^TM^)	Total (all devices)
(mean ± SD)	1.2 ± 0.4	2.1 ± 1.7	1.7 ± 1.4	1.4 ± 0.9	2.9 ± 3.0
First-pass rate of NeVa^TM^ ≥mTICI 2c	26/39 (66.7)				
First-line device	16/24 (66.7)				
Second-line device	10/15 (66.7)				
mTICI score	After NeVa^TM^ first-line	After NeVa^TM^ first-line + ADM	Before NeVa^TM^ second-line	After NeVa^TM^ second-line	Final
mTICI 3	14 (58.3)	20 (83.3)	0 (0.0)	8 (53.3)	29 (74.4)
mTICI 2c	4 (16.7)	4 (16.7)	1 (6.7)	4 (26.7)	7 (17.9)
mTICI 2b	4 (16.7)	0 (0.0)	0 (0.0)	1 (6.7)	1 (2.6)
mTICI 2a	1 (4.2)	0 (0.0)	2 (13.3)	0 (0.0)	0 (0.0)
mTICI 1	1 (4.2)	0 (0.0)	3 (0.2)	1 (6.7)	1 (2.6)
mTICI 0	0 (0.0)	0 (0.0)	9 (0.6)	1 (6.7)	1 (2.6)
mTICI ≥2b	22 (91.7)	24 (100)	1 (6.7)	13 (86.7)	37 (94.9)
mTICI ≥2c	18 (75.0)	24 (100)	1 (6.7)	12 (80.0)	36 (92.3.)
Hemorrhage on 24h post-thrombectomy dual-energy CT	13/39 (33.3)				
SICH	0/39 (0.0)				
In-hospital mortality	4/39 (10.3)				
NIHSS					
At admission	13.9 ± 6.8	0.695*			
24h after thrombectomy	13.1 ± 12.1				
At discharge	11.2 ± 12.7				
mRS					
At admission	4.6 ± 0.8	0.539*			
At discharge	3.3 ± 2.0				
mRS 0–2 at discharge	14/39 (35.9)				

Abbreviations: *ADM* additional maneuvers; *mRS* modified Rankin scale; *mTICI* modified thrombolysis in cerebral infarction; *NIHSS* National Institutes of Health Stroke Scale; *No* number; *SD*: standard deviation; *SICH* symptomatic intracerebral hemorrhage; *p-values are given for binary logistic regression for respective baseline parameters regarding first-pass recanalization mTICI≥2c with NeVa^TM^ stent retriever

When NeVa^TM^ stent retriever was used as first-line device (24/39 patients), a mean of 1.2±0.4 passes was performed. Here, a first-pass rate mTICI ≥ 2c of 66.7% (16/24 patients) was observed. After first-line use of NeVa^TM^ stent retriever, a rate of mTICI≥ 2b of 91.7% (22/24) and of mTICI≥2c of 18/24 (75%) was achieved. Successful recanalization ≥ mTICI 2c was achieved in all (16/16, 100%) procedures, where only the NeVa^TM^ stent retriever and no other device has been used.

Additional maneuvers with a different stent retriever to further improve recanalization rate were performed at the discretion of the performing neurointerventionalist in 7/24 (29.1%) patients with a mean of 1.7±0.9 additional passes. Those additional stent retrievers were mainly used for the remaining peripheral vessel occlusions (≥ distal M2) after partial recanalization (4/7, 57.1%), while they were used for remaining main vessel occlusion in 2/7 (28.6%) and in 1/7 (14.2%) for peripheral vessel occlusion (A3) due to embolization to new territory. Final mTICI rate after first-line NeVa^TM^ stent retriever and additional maneuvers was mTICI ≥ 2c of 100% (24/24).

NeVa^TM^ stent retriever was used as second-line device for mechanical thrombectomy in 15/39 (38.5%) patients. A mean number of 2.1±1.7 passes were performed with other stent retrievers afore NeVa^TM^ stent retriever was used. Here, in 6/15 patients a pRESET 6 × 50 mm (phenox, Bochum, Germany) was used before usage of NeVa^TM^ stent retriever, while in 3/15 patients a pRESET 6 × 50 mm and a CatchView 5.5 × 50 mm (balt, Montmorency, France), in 2/15 patients a CatchView 5.5 × 50 mm, in 1/15 patient a pRESET 6 × 50 mm and a pRESET 4 × 20 mm, in 1/15 patient a pRESET 6 × 30 mm, in 1/15 patients a pRESET 6 × 30 and a CatchView 5.5 × 50 mm, and in 1/15 patients a pRESET 4 × 20 mm stent retriever was used. Before usage of NeVa^TM^ stent retriever, the rate of mTICI ≥2b/2c was 6.7% (1/15, second-line NeVa^TM^ stent retriever was used for remaining M2 in this case) with all other patients showing mTICI ≤ 2a (mTICI 0: 9; mTICI 1: 3; mTICI 2a: 2).

A mean of 1.7±1.4 passes with NeVa^TM^ stent retriever as second-line device was performed, showing a first-pass rate mTICI ≥2c of 66.7% (10/15 patients). Herewith, the rate of mTICI ≥2b improved to 86.7 % (13/15) and of mTICI ≥ 2c to 80% (12/15). The two patients still showing mTICI <2b after second-line NeVa^TM^ maneuvers were both also not recanalizable with any other additional maneuvers/devices (final mTICI score 0 and 1, respectively). No embolization to new territory was observed in the second-line group.

Summarizing the entire cohort (39 patients) with first- or second-line use of NeVa^TM^ stent retriever, a total of 2.9±3.0 passes (median 2, range 1–16), of which 1.4±0.9 passes (median 1, range 1-5) were with NeVa^TM^ stent retriever, were performed. NeVa^TM^ stent retriever first-pass rate mTICI ≥2c was 66.7% (26/39). After thrombectomy with NeVa^TM^ stent retriever first- or second-line and additional maneuvers, a rate of mTICI ≥2b of 94.9% (37/39) and of mTICI ≥2c of 92.3% (36/39) was observed.

There were neither any baseline patient characteristics (age, sex, mRS, NIHSS, location, side or number of vessel occlusions, etiology of stroke) nor any procedural parameters (systemic i.v. lysis prior to thrombectomy, use of balloon-guided or intermediate catheter, NeVa^TM^ stent retriever as first- or second-line device) significantly associated with a successful first-pass recanalization of the NeVa^TM^ stent retriever (all *p*-values >0.05).

### Mechanical thrombectomy: safety evaluation

No minor or major adverse events were observed in the study cohort. Only 1 patient (2.6%) showed NeVa^TM^ stent retriever-associated intracranial vasospasm after thrombectomy, which fully resolved after short increase in running rate of catheter flushing solution containing nimodipine. A total of 13/39 (33.3%) patients showed a hemorrhage at 24h postprocedural CT scan, of which none was a symptomatic intracerebral hemorrhage (SICH) as defined by worsening of the NIHSS of at least four points. In these cases, vessel occlusion was located in 9/13 (69.2%) within the M1-segment, in 3/13 (23.1%) patients within the M2-segment and in 1/13 (7.7%) within the ICA and M1-segment. We have added this information to the results section of the manuscript. Overall in-hospital mortality rate was 4/39 (10.3%) patients.

### Clinical outcome evaluation

NIHSS score showed significant differences at admission, 24h after MT, and at discharge as revealed by the Friedman test (X^2^=22.4, *p*<0.001). Here, Friedman post hoc test showed that NIHSS was significantly lower at 24h after MT (13.1±12.1, *p*=0.008) and at discharge (11.2±12.7, *p*<0.001) than at admission (13.9±6.8). Meanwhile, NIHSS was not significantly different at 24h after MT compared to discharge (*p*=0.141). mRS improved significantly from 4.6±0.8 at admission to 3.3±2.0 at discharge (*p*<0.001). mRS 0–2 was reached in 14/39 (35.9%) of patients at discharge.

## Discussion

This study shows high technical effectiveness and safety profile of the NeVa^TM^ stent retriever for mechanical thrombectomy in acute ischemic stroke. In detail, the first-pass rate (recanalization ≥ mTICI 2c) of the NeVa^TM^ stent retriever was observed with 66.7%, which is higher than reported for other stent retriever devices (~22–40%) [23–28]. Since successful first-pass recanalization is associated with a better and an increasing number of passes is associated with a worse outcome [9, 23], this is an important device-related measure most probably explainable by the high radial force and the drop-zone design of the NeVa^TM^ stent retriever. First-pass rate was also higher than reported for the initial experiences with the NeVa^TM^ stent retriever reporting a first-pass rate of 43.9–48.3% [15–17, 19] and more similar to the up to date largest cohort reporting a rate of 53.8% in first-line treatment with NeVa^TM^ stent retriever [18]. To the best of our knowledge, this is the first study also reporting effectivity of the NeVa^TM^ stent retriever as second-line device. Here, first-pass rate was as well 66.7% after various other stent retrievers failed to recanalize after a mean of 2.1±1.7 passes emphasizing the potential of the NeVa^TM^ stent retriever as an effective rescue device. This notion should be further evaluated in future prospective studies including a larger cohort of patient with second-line use of the NeVa^TM^ stent retriever.

On the other hand, our study reports the need for other devices after first-line NeVa^TM^ stent retriever in 29.1% of cases, which is higher (7.6–13.8%) [15, 16], than reported in other studies on other devices. This is best explained due to a lower number of passes performed with NeVa^TM^ stent retriever especially when used as first-line device compared to elsewhere indicating an earlier decision towards a change of device [15–17]. Further, this can be explained by a high rate (57.1%) of other devices needed only for remaining peripheral vessel occlusions (≥distal M2). However, a contribution of partial clot retraction with consequent distal emboli cannot be fully excluded here.

Nevertheless, a preferably high recanalization rate should be the goal of MT also beyond the first-pass effect [26]. Here, final recanalization mTICI ≥2b including rescue strategies was with 94.9% also higher than with other studies using NeVa^TM^ [15–17], or other stent retrievers, for, e.g., of 80.3% in the Trevo (Stryker, Kalamazoo, USA), Stent Retriever Acute Stroke (TRACK) multicenter registry [29], of 85.3% for the Aperio stent retriever (Acandis, Pforzheim, Germany) [30], or 88% for the Solitaire (Medtronic, Irvine, USA) stent retriever in the SWIFT Prime study [4]. However, a recent study on NeVa^TM^ stent retriever first-line use reported a final mTICI ≥2b of 94.2% similar to our data [18]. Importantly, our data shows that a considerably high amount of patients (86.7%) improved in final recanalization up to a mTICI ≥2b when NeVa^TM^ stent retriever was used as second-line stent retriever again showing the potential of this device also as rescue strategy. Additionally, it has been shown that larger stent retrievers may improve first-pass rate and final mTICI score [11, 12]. In this context, the 4 × 30 mm NeVa^TM^ stent retriever evaluated here is a rather small device. Importantly, NeVa^TM^ devices are available up to 4.5 × 44 mm with 5 drop zones potentially even increasing recanalization rates (but maybe also potential complications), which will have to be evaluated in future studies.

Regarding the safety profile, our study did not find any device-related procedural complications, similarly to previous studies reporting adverse events only very rarely [15–18]. A previous study reported a high rate (48.3%) of vasospasms in the recanalized segment after thrombectomy with NeVa^TM^ stent retriever [16]. Other studies on the NeVa^TM^ device did not observe or explicitly report on such a high rate of vasospasm [15, 17, 18]. Our data observed intracranial vasospasms only in 2.6% which is similar to the rate reported in most studies with other thrombectomy devices [27, 31]. This might be explained by the difference in procedural technique, since Borggrefe et al. report to have only temporarily connected the catheter to a continuous flush line charged with nimodipine in case of observed vasospasms [16], while such nimodipine charged flush line is always connected to the guiding catheter at our center potentially preventing from such a high rate of periprocedural vasospasms. Meanwhile, we did observe a comparably high rate of intracranial hemorrhages at 24h after MT (33.3%). However, none of these was symptomatic confirming the low rate of SICH after usage of the NeVa^TM^ stent retriever observed in previous studies [15–17]. Thus, NeVa^TM^ stent retriever proofed as safe device for MT in our study cohort. However, one has to consider the high mechanical traction forces of this device implying the risk for complications such as caroticocavernous fistulas as reported elsewhere [16].

The above-mentioned recanalization rates resulted in a mean ± SD mRS of 3.3±2.0 and a rate of 35.9% with mRS 0–2 at discharge which is, considering the relatively small study cohort, comparable to a previous report (4.0±1.7 or 24%, respectively) [16] but lower than commonly reported after 90 days [15].

### Limitations

This study is limited by its retrospective, single-center, and self-reported design and the relatively small number of patients included. Further, the study cohort has some heterogeneity regarding first- or second-line usage of the NeVa^TM^ stent retriever, usage of balloon-guided or intermediate catheters, or variety in rescue strategies. Further, due to the lack of evidence-based recommendations, the use of the NeVa^TM^ stent retriever as first- or second-line device was left to the discretion of the performing neurointerventionalist, thus leading to a potential selection bias. However, with this design, the study cohort represents a “real-world” dataset for the applicability and performance of the tested stent retriever. Further, this study does not include long-term clinical follow-up as the primary criteria were technical efficacy and safety profile. Future prospective and comparative studies in larger cohorts are needed to further characterize the optimal setting and limitations when to use the NeVa^TM^ stent retriever as well as to compare long-term clinical outcome with those of other devices.

In conclusion, this study shows a high technical effectiveness and good safety profile of the NeVa^TM^ stent retriever as first- and second-line device for mechanical thrombectomy in acute ischemic stroke.
